# The global convergence of some self-scaling conjugate gradient methods for monotone nonlinear equations with application to 3DOF arm robot model

**DOI:** 10.1371/journal.pone.0317318

**Published:** 2025-01-24

**Authors:** Sulaiman M. Ibrahim, Lawal Muhammad, Rabiu Bashir Yunus, Muhammad Yusuf Waziri, Saadi bin Ahmad Kamaruddin, Aceng Sambas, Nooraini Zainuddin, Ali F. Jameel

**Affiliations:** 1 Institute of Strategic Industrial Decision Modelling, School of Quantitative Sciences, Universiti Utara Malaysia, Sintok, Kedah, Malaysia; 2 Faculty of Education and Arts, Sohar University, Sohar, Oman; 3 Department of Mathematics, Faculty of Science, Northwest University Kano, Kano, Nigeria; 4 Department of Mathematics, Faculty of Computing and Mathematical Sciences, Kano University of Science and Technology, Wudil, Nigeria; 5 Department of Fundamental and Applied Sciences, Faculty of Science and Information Technology, Universiti Teknologi PETRONAS, Bandar Seri Iskandar, Perak Darul Ridzuan, Malaysia; 6 Faculty of Physical Sciences, Bayero University Kano, Kano, Nigeria; 7 Faculty of Information and Computing, Universiti Sultan Zainal Abidin, Terengganu, Malaysia; 8 Department of Mechanical Engineering, Universitas Muhammadiyah Tasikmalaya, Tasikmalaya, Indonesia; CINVESTAV IPN: Centro de Investigacion y de Estudios Avanzados del Instituto Politecnico Nacional, MEXICO

## Abstract

Conjugate Gradient (CG) methods are widely used for solving large-scale nonlinear systems of equations arising in various real-life applications due to their efficiency in employing vector operations. However, the global convergence analysis of CG methods remains a significant challenge. In response, this study proposes scaled versions of CG parameters based on the renowned Barzilai-Borwein approach for solving convex-constrained monotone nonlinear equations. The proposed algorithms enforce a sufficient descent property independent of the accuracy of the line search procedure and ensure global convergence under appropriate assumptions. Numerical experiments demonstrate the efficiency of the proposed methods in solving large-scale nonlinear systems, including their applicability to accurately solving the inverse kinematic problem of a 3DOF robotic manipulator, where the objective is to minimize the error in achieving a desired trajectory configuration.

## 1 Introduction

Conjugate gradient (CG) methods are among the efficient family of optimization algorithms, particularly suited for large-scale nonlinear problems [1–3]. These methods are often defined to solve problems of the form:

$$\begin{array}{c} F\left(x\right)=0\;x\in R^{n}, \end{array}$$ (1)

where $F:R^{n}\to R^{n}$ is a continuous and monotone function, and $R^{n}$ is nonempty, closed, and convex. The monotonicity of *F* implies that

$$\begin{array}{c} {\left(F\left(x\right)-F\left(y\right)\right)}^{T}\left(x-y\right)\geq 0,\;x,y\in R^{n}. \end{array}$$ (2)

The general CG algorithm is designed to solve the constrained problem (1) via the following iterative formula;

$$\begin{array}{c} x_{k+1}=x_{k}+\alpha_{k}d_{k}, \end{array}$$ (3)

where *α*_*k*_ > 0 is the step length which is obtained using a suitable line search strategy and *d*_*k*_ is the search direction generated by:

$$\begin{array}{c} d_{k}=\{\begin{array}{c} -F_{k}\;\;\;\;\;if\;k=1\\ -F_{k}+\beta_{k}d_{k-1}\;if\;k\geq 2. \end{array} \end{array}$$ (4)

From the search direction, the coefficient *β*_*k*_ is a scalar termed as CG parameter and *F*_*k*_ = *F*(*x*_*k*_) defines the function value of *F* at *x*_*k*_. The choices of *β*_*k*_ influence the of any CG method and thus, many researchers have studied effective choices of *β*_*k*_ (see; [4–24]).

Among the modifications available in the literature, the Riavie-Mamat-Ismail-Leong (RMIL) [25] is considered one of the effective CG coefficients with a restart feature. Rivaie et al. [25] constructed the RMIL formula as follows:

$$\begin{array}{c} \beta_{k}^{RMIL}=\frac{F_{k+1}^{T}y_{k}}{\left|\right|d_{k}{\left|\right|}^{2}}. \end{array}$$ (5)

The author further presented the modification of RMIL called MRMIL [26] with a formula defined as follows:

$$\begin{array}{c} \beta_{k}^{MRMIL}=\frac{F_{k+1}^{T}\left(F_{k+1}-F_{k}-d_{k}\right)}{\left|\right|d_{k}{\left|\right|}^{2}}, \end{array}$$ (6)

and proved the global convergence of the method under suitable assumptions. Results from numerical experiments demonstrate the efficiency of the MRMIL method on a set of unconstrained optimization problems. Motivated by the nice convergence features of the RMIL methods, several researchers have developed other versions of the formula (see; [27–32]).

Previous studies have shown that advanced optimization techniques can significantly enhance the performance of robotic systems. For instance, Kim et al., [33] introduced a class of Newton-type algorithms for optimizing robot motions that consider dynamics. This allows for the formulation of the exact gradient and Hessian of a dynamics-involved objective function, leading to efficient second-order Newton-type optimization algorithms for optimal robot motion generation. In the context of robotic control, Yoshikawa [34] highlighted the importance of accurate dynamic modeling and optimization for achieving precise and efficient robot movements. Lee et al., [35] describe Newton and quasi-Newton minimization procedures for dynamics-based robot movement generation by modelling the considered robot as rigid multi-body systems containing redundant actuators and sensors, active and passive joints, and multiple closed loops. Although it is theoretically possible to derive the equations of motion for such systems analytically, their complexity renders classical optimization methods impractical for anything but the simplest systems. Specifically, numerical approximation of the gradient and Hessian frequently results in ill-conditioning and poor convergence.

Recently, Yahaya et al., [36, 37] constructed some quasi-Newton schemes to meet the requirements of the weak secant equation. These methods are further extended to solve the 3DOF planar robot arm manipulator. All these methods are based on Newton and Quasi-Newton updates. Studies have shown that the Newton method faces many challenges when employed to solve large-scale problems. This is because it is nearly impossible to compute the Jacobian matrix exactly in each iteration. On the other hand, the quasi-Newton methods can inherit some appropriate theoretical and numerical properties from the Newton method since they approximate the Jacobian matrix [38]. However, its matrix operations can also be computationally expensive when solving large-scale problems, thus, the quasi-Newton approaches are also not as effective in handling these kinds of issues.

The CG methods appear to be an appropriate alternative for handling large-scale problems since their algorithms solely employ vector operations. Betts [39] in their work on trajectory optimization, demonstrated the efficiency of CG methods in solving large-scale problems in aerospace applications. Similarly, Nocedal and Wright [40] provided a comprehensive overview of optimization methods, including CG, emphasizing their applicability to a wide range of engineering problems. However, as previously stated, the global convergence analysis of CG methods is typically not easy to achieve. Hence, motivated by the above discussion and by exploring the advantages of the RMIL conjugate gradient procedure, the Quasi-Newton direction, the Quasi-Newton equation, and the famous Barzilai-Borwein strategy, the study proposed a family of scaled CG parameters for a monotone nonlinear system of equations. The main features of the new algorithms are as follows:

- Introducing two self-scaling matrix-free CG algorithms based on the famous Barzilai-Borwein strategy.
- Show that the proposed methods enforce a sufficient descent property irrespective of the line search procedure.
- Prove the global convergence of the new methods using monotonicity condition and Lipschitz continuous of the function *F*.
- Demonstrate practical implementation of the proposed methods for trajectory planning and real-time control in the 3DOF arm robot. This will highlight the benefits of self-scaling CG algorithms for complex robotic systems.
- Comparing the efficiency and robustness of existing CG algorithms in terms of residual Error and computational efficiency.

The study’s remaining sections are organized as follows. The suggested algorithms utilizing the scaling strategy will be covered in the following Section, and Section 3 presents the method’s global convergence under appropriate assumptions. Section 4 will present the numerical experiments, and Section 5 will include the conclusion.

## 2 A scaled RMIL and MRMIL CG algorithms

This section will present scale RMIL and MRMIL CG algorithms for monotone nonlinear equations. The RMIL and MRMIL CG parameters are given by

$$\begin{array}{c} \beta_{k}^{RMIL}=\frac{F_{k+1}^{T}y_{k}}{\left|\right|d_{k}{\left|\right|}^{2}}, \end{array}$$ (7)

and MRMIL [26] with the following formula:

$$\begin{array}{c} \beta_{k}^{MRMIL}=\frac{F_{k+1}^{T}\left(F_{k+1}-F_{k}-d_{k}\right)}{\left|\right|d_{k}{\left|\right|}^{2}}. \end{array}$$ (8)

The scaling strategy has been an effective procedure for enhancing the theoretical performances of the CG algorithms, see [41–44]. In line with these developments, we proposed a scale RMIL and MRMIL CG parameters as

$$\begin{array}{c} \beta_{k}^{SRMIL}=\gamma_{k}\beta_{k}^{RMIL}, \end{array}$$ (9)

and

$$\begin{array}{c} \beta_{k}^{SMRMIL}=\lambda_{k}\beta_{k}^{MRMIL}, \end{array}$$ (10)

such that |*γ*_*k*_| ≤ 1 and |λ_*k*_| ≤ 1. Gilbert and Nocedal [45] defined a new CG formula and show that any CG method with parameter $\beta_{k}=\gamma_{k}\beta_{k}^{FR}$ in which |*γ*_*k*_| ≤ 1, satisfies the SDC [46]. The primary contribution of this study is determining the value of the scalars *γ*_*k*_ and λ_*k*_ at each iteration.

### 2.1 Computing the scaling parameter based on Barzilai-Borwein approach

This section constructs the optimal scaled choice parameter *γ*_*k*_ that incorporates some important features of the famous Barzilai-Borwein (BB) [47] procedure including the spectral parameters whose formulas are defined as as

$$\begin{array}{c} \lambda_{k}^{1}=\frac{s_{k}^{T}s_{k}}{y_{k}^{T}s_{k}}\;\text{and}\;\lambda_{k}^{2}=\frac{s_{k}^{T}y_{k}}{y_{k}^{T}y_{k}}. \end{array}$$ (11)

Now, considering Perry’s point of view [48], the scaled RMIL and MRMIL directions can be obtained as

$$\begin{array}{c} d_{0}=-F_{0},\;\;\;d_{k+1}=-A_{k+1}F_{k+1},\;k\geq 0, \end{array}$$ (12)

where *A*_*k*+1_ is the search direction matrix defined by

$$\begin{array}{c} A_{k+1}=I-\gamma_{k}\frac{y_{k}d_{k}^{T}}{\left|\right|d_{k}{\left|\right|}^{2}}. \end{array}$$ (13)

By taking advantage of the BB approach [47], the study constructed the formula for *γ*_*k*_ as the solution to the minimization problem of the form:

$$\begin{array}{c} \underset{\gamma }{\text{min}}{∥A_{k+1}-\lambda_{k}I∥}_{F}^{2}, \end{array}$$ (14)

with ‖.‖_*F*_ representing the Frobenius matrix norm and

$$\begin{array}{c} \lambda_{k}=\text{max}\{\lambda_{\text{min}},\text{min}\{\lambda_{k}^{i},\lambda_{\text{max}}\}\},\;i=1,2, \end{array}$$ (15)

where the 0 < λ_min_ < λ_max_ < ∞. If we assigned *M* = *A*_*k*+1_ − *λI* and using the relation ${∥M∥}_{F}^{2}=Trace\left(M^{T}M\right)$, then

$$\begin{array}{c} M_{k+1}=\left(1-\lambda_{k}I\right)-\gamma_{k}\frac{y_{k}d_{k}^{T}}{\left|\right|d_{k}{\left|\right|}^{2}}, \end{array}$$

$$\begin{array}{c} M_{k+1}^{T}=\left(1-\lambda_{k}I\right)-\gamma_{k}\frac{d_{k}y_{k}^{T}}{\left|\right|d_{k}{\left|\right|}^{2}}, \end{array}$$

$$\begin{array}{c} M_{k+1}^{T}M_{k+1}={\left(1-\lambda_{k}\right)}^{2}I-\left(1-\lambda_{k}\right)\gamma_{k}\frac{y_{k}d_{k}^{T}}{\left|\right|d_{k}{\left|\right|}^{2}}-\left(1-\lambda_{k}\right)\gamma_{k}\frac{d_{k}y_{k}^{T}}{\left|\right|d_{k}{\left|\right|}^{2}}+\gamma_{k}^{2}\frac{d_{k}y_{k}^{T}y_{k}d_{k}^{T}}{\left|\right|d_{k}{\left|\right|}^{4}} \end{array}$$

$$\begin{array}{c} Tr\left(M_{k+1}^{T}M_{k+1}\right)={\left(1-\lambda_{k}\right)}^{2}n-2\gamma_{k}\left(1-\lambda_{k}\right)\frac{y_{k}^{T}d_{k}}{\left|\right|d_{k}{\left|\right|}^{2}}+\gamma_{k}^{2}\frac{\left|\right|y_{k}{\left|\right|}^{2}\left|\right|d_{k}{\left|\right|}^{2}}{\left|\right|d_{k}{\left|\right|}^{4}}. \end{array}$$ (16)

Differentiating this with respect to *γ*_*k*_ and equating it to zero will produce:

$$\begin{array}{c} 2\left(1-\lambda_{k}\right)\frac{y_{k}d_{k}^{T}}{\left|\right|d_{k}{\left|\right|}^{2}}=2\gamma_{k}\frac{\left|\right|y_{k}{\left|\right|}^{2}\left|\right|d_{k}{\left|\right|}^{2}}{\left|\right|d_{k}{\left|\right|}^{4}}, \end{array}$$ (17)

solving for *γ*_*k*_, we obtained the following solution for (14) as

$$\begin{array}{c} \gamma_{k}=\left(1-\lambda_{k}\right)\frac{d_{k}^{T}y_{k}}{{∥y_{k}∥}^{2}}. \end{array}$$ (18)

We suggested the following revised form of (18), satisfying $|\gamma_{k}^{1}|\leq 1.$

$$\begin{array}{c} \gamma_{k}^{1}=min\{1,|\gamma_{k}\left|\right\}. \end{array}$$ (19)

In a similar approach we can determine the scaling parameter in (10) using (8) as follows;

Let us define

$$\begin{array}{c} d_{k+1}=-B_{k+1}F_{k+1}, \end{array}$$ (20)

where the search direction matrix is define by

$$\begin{array}{c} B_{k+1}=I-\lambda_{k}\frac{\left(F_{k+1}-F_{k}-d_{k}\right)d_{k}^{T}}{\left|\right|d_{k}{\left|\right|}^{2}}. \end{array}$$

The study also constructed the formula for λ_*k*_ as the solution to the minimization problem of the form:

$$\begin{array}{c} \underset{\lambda }{\text{min}}{∥B_{k+1}-\gamma_{k}I∥}_{F}^{2}, \end{array}$$ (21)

with ‖.‖_*F*_ representing the Frobenius matrix norm and

$$\begin{array}{c} \gamma_{k}=\text{max}\{\gamma_{\text{min}},\text{min}\{\gamma_{k}^{i},\gamma_{\text{max}}\}\},\;i=1,2, \end{array}$$ (22)

where the 0 < *γ*_min_ < *γ*_max_ < ∞

Let

$$\begin{array}{c} N_{k+1}=\left(1-\gamma_{k}\right)I-\lambda_{k}\frac{\left(F_{k+1}-F_{k}-d_{k}\right)d_{k}^{T}}{\left|\right|d_{k}{\left|\right|}^{2}}, \end{array}$$

$$\begin{array}{c} N_{k+1}^{T}=\left(1-\gamma_{k}\right)I-\lambda_{k}\frac{d_{k}{\left(F_{k+1}-F_{k}-d_{k}\right)}^{T}}{\left|\right|d_{k}{\left|\right|}^{2}}, \end{array}$$

$$\begin{array}{cc} N_{k+1}^{T}N_{k+1}= & \;{\left(1-\gamma_{k}\right)}^{2}I-\left(1-\gamma_{k}\right)\lambda_{k}\frac{d_{k}{\left(F_{k+1}-F_{k}-d_{k}\right)}^{T}}{\left|\right|d_{k}{\left|\right|}^{2}}\\ & -\left(1-\gamma_{k}\right)\lambda_{k}\frac{d_{k}{\left(F_{k+1}-F_{k}-d_{k}\right)}^{T}}{\left|\right|d_{k}{\left|\right|}^{2}}+\lambda_{k}^{2}\frac{\left|\right|\left(F_{k+1}-F_{k}-d_{k}\right){\left|\right|}^{2}d_{k}d_{k}^{T}}{\left|\right|d_{k}{\left|\right|}^{4}}. \end{array}$$ (23)

$$\begin{array}{cc} Tr(N_{k+1}^{T}N_{k+1})= & \;{\left(1-\gamma_{k}\right)}^{2}n-2\left(1-\gamma_{k}\right)\lambda_{k}\frac{d_{k}^{T}\left(F_{k+1}-F_{k}-d_{k}\right)}{\left|\right|d_{k}{\left|\right|}^{2}}\\ & +\lambda_{k}^{2}\frac{\left|\right|\left(F_{k+1}-F_{k}-d_{k}\right){\left|\right|}^{2}\left|\right|d_{k}{\left|\right|}^{2}}{\left|\right|d_{k}{\left|\right|}^{4}}. \end{array}$$ (24)

Differentiating with respect to λ_*k*_ and settting the result equal to zero we obtained

$$\begin{array}{c} -2\left(1-\gamma_{k}\right)\frac{d_{k}^{T}\left(F_{k+1}-F_{k}-d_{k}\right)}{\left|\right|d_{k}{\left|\right|}^{2}}+2\lambda_{k}\frac{\left|\right|\left(F_{k+1}-F_{k}-d_{k}\right){\left|\right|}^{2}}{\left|\right|d_{k}{\left|\right|}^{2}}=0. \end{array}$$

Solving for λ_*k*_ we obtained the following MRMIL scaling parameter

$$\begin{array}{c} \lambda_{k}=\left(1-\gamma_{k}\right)\frac{d_{k}^{T}\left(F_{k+1}-F_{k}-d_{k}\right)}{\left|\right|\left(F_{k+1}-F_{k}-d_{k}\right){\left|\right|}^{2}}, \end{array}$$ (25)

and suggested the following revised form of (25), satisfying $|\lambda_{k}^{*}|\leq 1$:

$$\begin{array}{c} \lambda_{k}^{*}=min\{1,|\lambda_{k}\left|\right\}. \end{array}$$ (26)

Additionally, to guarantee that the sufficient descent condition is fulfilled regardless of the line search method, the study defined the scaled RMIL and MRMIL CG directions by

$$\begin{array}{c} d_{k+1}=-\hat{\zeta_{k}}F_{k+1}+\gamma_{k}^{1}\beta_{k}^{RMIL}d_{k},\;k=0,1,\cdots , \end{array}$$ (27)

and

$$\begin{array}{c} d_{k+1}=-\zeta_{k}^{3}F_{k+1}+\lambda_{k}^{*}\beta_{k}^{MRMIL}d_{k},\;k=0,1,\cdots , \end{array}$$ (28)

where

$$\begin{array}{c} \hat{\zeta_{k}}=1+\gamma_{k}^{1}\frac{\beta_{k}^{RMIL}F_{k+1}^{T}d_{k}}{\left|\right|F_{k+1}{\left|\right|}^{2}},\;\zeta_{k}^{3}=1+\lambda_{k}^{*}\frac{\beta_{k}^{MRMIL}F_{k+1}^{T}d_{k}}{\left|\right|F_{k+1}{\left|\right|}^{2}}. \end{array}$$ (29)

**Algorithm 1:** The scaled RMIL projection-based CG algorithm (SRMILCG)

**Step 1**: Input $x_{0}\in R^{n},\epsilon >0,$ and *σ* ∈ (0, 1), *μ* ∈ (0, 1), *ν* > 0, *κ* > 0, 0 < *γ*_min_ < *γ*_max_ and by setting *k* = 0 and computing *d*_0_ = −*F*_0_.

**Step 2**: If ||*F*_*k*_|| ≤ *ϵ*, stop; if not, move on to Step 3.

**Step 3**: Obtain the *d*_*k*_ via (27) or (28), with *y*_*k*_ = *F*(*w*_*k*_) − *F*_*k*_ + *σs*_*k*_ and *s*_*k*_ = *v*_*k*_ − *x*_*k*_.

**Step 4**: Set *w*_*k*_ = *x*_*k*_ + *α*_*k*_*d*_*k*_ and calculate *α*_*k*_ = *max*{*νμ*^*i*^ *i* = 1, 2, 3…} to satisfy

$$\begin{array}{c} -F{\left(v_{k}\right)}^{T}d_{k}\geq \kappa \alpha_{k}||F\left(v_{k}\right)\left|||\right|d_{k}{\left|\right|}^{2} \end{array}$$ (30)

**Step 5** If *w*_*k*_ ∈ *ψ* and ||*F*(*w*_*k*_)|| = 0 stop, otherwise

$$\begin{array}{c} x_{k+1}=P_{\psi }\left(x_{k}-z_{k}F\left(h_{k}\right)\right), \end{array}$$ (31)

where *P* is the projection operator, and

$$\begin{array}{c} z_{k}=\frac{F{\left(w_{k}\right)}^{T}\left(x_{k}-z_{k}\right)}{||F\left(w_{k}\right){\left|\right|}^{2}} \end{array}$$ (32)

**Step 6**: Proceed to step 2 after setting that *k* = *k* + 1.

## 3 Convergence analysis

In this section, we will show the global convergence of the scaled RMIL algorithm, assuming that *F* is monotonic and Lipschitz continuous.

**Lemma 3.1** The line search in (30) is well-defined for all *k* ≥ 0.

Proof: Assume there exists *k*_0_ ≥ 0 under the condition that for *i* = 0, 1, 2, …

$$\begin{array}{c} -F{\left(x_{k_{0}}+\nu \mu^{i}d_{k_{0}}\right)}^{T}d_{k_{0}}<\kappa \nu \mu^{i}||F\left(x_{k_{0}}+\nu \mu^{i}d_{k_{0}}\right)\left|||\right|d_{k_{0}}{\left|\right|}^{2}. \end{array}$$ (33)

Let *i* → ∞ in (33), then,

$$\begin{array}{c} F{\left(x_{k_{0}}\right)}^{T}d_{k_{0}}\leq 0. \end{array}$$ (34)

Since *d*_*k*_ defined by (27) or (28) satisfy the SDC, it follows that:

$$\begin{array}{c} -F{\left(x_{k_{0}}\right)}^{T}d_{k_{0}}\geq ||F\left(x_{k_{0}}\right){\left|\right|}^{2}>0. \end{array}$$ (35)

Hence, Eqs (34) and (35) lead to a contradiction, thereby completing the proof.

**Lemma 3.2** [44] If the sequence {*x*_*k*_} and {*u*_*k*_} are given by the SRMILCG method, then, for certain *M* > 0, it implied:

$$\begin{array}{c} \left|\right|F_{k}\left|\right|\leq D,\;\underset{k\to \infty }{\text{lim}}\alpha_{k}\left|\right|d_{k}\left|\right|=0,\text{for}\;\text{all}\;k. \end{array}$$ (36)

proof: From line search (30), we get:

$$\begin{array}{c} F{\left(w_{k}\right)}^{T}\left(x_{k}-w_{k}\right)=-F{\left(w_{k}\right)}^{T}d_{k}\geq J\alpha_{k}^{2}||F\left(w_{k}\right)\left|||\right|d_{k}{\left|\right|}^{2}>0. \end{array}$$ (37)

Let x^*^ ∈ *R*^*n*^ satisfying *F*(x^*^) = 0, by the monotonicity of *F*, it implies:

$$\begin{array}{c} F{\left(w_{k}\right)}^{T}\left(x_{k}-x^{*}\right)=F{\left(w_{k}\right)}^{T}\left(x_{k}-w_{k}\right)+F{\left(w_{k}\right)}^{T}\left(w_{k}-x^{*}\right) \end{array}$$

$$\begin{array}{c} \geq F{\left(w_{k}\right)}^{T}\left(x_{k}-w_{k}\right)+F{\left(x^{*}\right)}^{T}\left(w_{k}-x^{*}\right) \end{array}$$ (38)

$$\begin{array}{c} =F{\left(w_{k}\right)}^{T}\left(x_{k}-w_{k}\right). \end{array}$$ (39)

Utilizing (37) and (38) to get:

$$\begin{array}{c} \left|\right|x_{k+1}-x_{k}^{*}{\left|\right|}^{2}=\left|\right|B_{\psi }\left(x_{k}-z_{k}F\left(w_{k}\right)\right)-x^{*}{\left|\right|}^{2}\\ \leq \left|\right|x_{k}-z_{k}F\left(w_{k}\right)-x^{*}{\left|\right|}^{2}\\ =\left|\right|x_{k}-x^{*}{\left|\right|}^{2}-2z_{k}F{\left(w_{k}\right)}^{T}\left(x_{k}-x^{*}\right)+z_{k}^{2}||F\left(w_{k}\right){\left|\right|}^{2}. \end{array}$$ (40)

By using the Cauchy Schwartz inequality in (40) and the definition of *z*_*k*_, we obtain:

$$\begin{array}{c} \left|\right|x_{k+1}-x^{*}{\left|\right|}^{2}\leq \left|\right|x_{k}-x^{*}{\left|\right|}^{2}-2z_{k}F{\left(w_{k}\right)}^{T}\left(x_{k}-w_{k}\right)+z_{k}^{2}||F\left(w_{k}\right){\left|\right|}^{2}\\ \leq \left|\right|x_{k}-x^{*}{\left|\right|}^{2}-\frac{{\left(F{\left(w_{k}\right)}^{T}\left(x_{k}-z_{k}\right)\right)}^{2}}{||F\left(w_{k}\right){\left|\right|}^{2}}\\ \leq \left|\right|x_{k}-x^{*}{\left|\right|}^{2}-J^{2}\left|\right|x_{k}-w_{k}{\left|\right|}^{4}, \end{array}$$ (41)

hence, we get:

$$\begin{array}{c} \left|\right|x_{k+1}-x^{*}\left|\right|\leq \left|\right|x_{k}-x^{*}\left|\right|,\forall k\geq 0. \end{array}$$ (42)

Thus, the sequence {||*x*_*k*_ − x^*^||} is decreasing and therefore convergent. Next, using (45) and the Lipschitz continuity of *F*, it follows that:

$$\begin{array}{c} ||F\left(x_{k}\right)\left|\right|=||F\left(x_{k}\right)-F\left(x^{*}\right)\left|\right|\leq L||x_{k}-x^{*}\left|\right|\leq L||x_{0}-x^{*}\left|\right|=D. \end{array}$$ (43)

By Cauchy Schwartz inequality, monotonicity of *F*, and (30), it follows that:

$$\begin{array}{c} \kappa ||F\left(w_{k}\right)\left|||\right|x_{k}-w_{k}{\left|\right|}^{2}\leq F{\left(w_{k}\right)}^{T}\left(x_{k}-w_{k}\right)\leq ||F\left(w_{k}\right)\left|||\right|x_{k}-w_{k}\left|\right|, \end{array}$$ (44)

which gives

$$\begin{array}{c} J||x_{k}-w_{k}\left|\right|\leq 1. \end{array}$$ (45)

We can observe that from (45) the sequence {*w*_*k*_} is bounded. Again, from (41), it follows that:

$$\begin{array}{c} J^{2}\sum_{k=0}^{\infty }\left|\right|x_{k}-w_{k}{\left|\right|}^{4}\leq \sum_{k=0}^{\infty }\left(|\right|x_{k}-x^{*}{\left|\right|}^{2}-\left|\right|x_{k+1}-x^{*}{\left|\right|}^{2})<\infty . \end{array}$$ (46)

This implies

$$\begin{array}{c} \underset{k\to \infty }{\text{lim}}\left|\right|x_{k}-w_{k}\left|\right|=\underset{k\to \infty }{\text{lim}}\alpha_{k}\left|\right|d_{k}\left|\right|=0, \end{array}$$ (47)

which completes the proof.

**Theorem 3.1** The proposed algorithm 1 demonstrates convergence, indicating that

$$\begin{array}{c} \underset{k\to \infty }{\text{lim}}\left|\right|F_{k}\left|\right|=0. \end{array}$$ (48)

Proof: Suppose that (49) does not hold true, i.e., for *r* > 0,

$$\begin{array}{c} \left|\right|F_{k}\left|\right|\geq r,\forall k\in S. \end{array}$$ (49)

Also from Lipschitz continuity of *F* it can be deduce that $\left|\right|y_{k}\left|\right|\leq \left(L+\kappa \right)||s_{k}\left|\right|=S,$ where L is the taken as the Lipschitz constant. Thus,

$$\begin{array}{c} \left|\right|d_{k+1}\left|\right|=∥-\tilde{\eta_{k}}F_{k+1}+\gamma_{k}^{1}\beta_{k}^{RMIL}d_{k},∥ \end{array}$$ (50)

$$\begin{array}{c} =∥\left(-1+\gamma_{k}^{1}\frac{\beta_{k}^{RMIL}F_{k+1}^{T}d_{k}}{\left|\right|F_{k+1}{\left|\right|}^{2}}\right)F_{k+1}+\gamma_{k}^{1}\beta_{k}^{RMIL}d_{k},∥ \end{array}$$ (51)

$$\begin{array}{c} \leq \left|\right|F_{k+1}\left|\right|+2\frac{\left|\right|F_{k+1}\left|||\right|y_{k}\left|\right|}{r^{2}}\left|\right|d_{k}\left|\right| \end{array}$$ (52)

$$\begin{array}{c} \leq D+2\frac{DS}{r^{2}}\left|\right|d_{k}\left|\right| \end{array}$$

Now, let $a=2\frac{DS}{\left|\right|r^{2}\left|\right|},$ therefore from (52) we have

$$\begin{array}{c} \left|\right|d_{k+1}\left|\right|\leq D+a||d_{k}\left|\right|\\ \leq D\left(1+a+a^{2}+\cdots +a^{k-k_{0}+1}\right)\left|\right|d_{k_{0}}\left|\right|\\ \leq \frac{D}{1-a}+\left|\right|d_{k_{0}}\left|\right| \end{array}$$ (53)

Hence, for $K=max\{||d_{1}||,||d_{2}||,\cdots ,||d_{k_{0}}||,\frac{D}{1-a}+||d_{k_{0}}||\},$ the boundedness of the proposed direction is achieved. Similarly, (28) can also be shown to be bounded. From the famous Cauchy-Schwartz inequality and sufficient condition, it follows that:

$$\begin{array}{c} \left|\right|F_{k}\left|||\right|d_{k}\left|\right|\geq -F_{k}d_{k}\geq \left|\right|F_{k}{\left|\right|}^{2}>0. \end{array}$$ (54)

From (54) we get

$$\begin{array}{c} \left|\right|d_{k}\left|\right|\geq \left|\right|F_{k}\left|\right|>r. \end{array}$$ (55)

Thus, From (47) and (55) we obtain

$$\begin{array}{c} \underset{k\to \infty }{\text{lim}}\;\alpha_{k}=0 \end{array}$$ (56)

Suppose there exists $\alpha_{k}^{\prime }$ not satisfying the search strategy (30), that is

$$\begin{array}{c} -F{\left(x_{k}+\alpha_{k}^{\prime }d_{k}\right)}^{T}d_{k}<\kappa \alpha_{k}^{\prime }||F\left(x_{k}+\alpha_{k}^{\prime }d_{k}\right)\left|||\right|d_{k}{\left|\right|}^{2}, \end{array}$$ (57)

Obviously, the sequence {*x*_*k*_} is bounded and has an infinite index set *J*_1_ and an accumulation point $\bar{x}$ satisfying ${\text{lim}}_{k\in J_{1}}x_{k}=\bar{x}.$ It also implies boundedness for ${\left\{d_{k}\right\}}_{k\in J_{1}}$. Consequently, an infinite index set *J*_2_ ⊂ *J*_1_ with an accumulation point $\bar{d}$ exists, and ${\text{lim}}_{k\in J_{2}}d_{k}=\bar{d}$ is satisfied by it.

After that, if we use the limit in (57), then,

$$\begin{array}{c} -F{\left(\bar{x}\right)}^{T}\tilde{d}\leq 0. \end{array}$$ (58)

once more putting a limit on (54)

$$\begin{array}{c} -F{\left(\bar{x}\right)}^{T}\tilde{d}\geq 0. \end{array}$$ (59)

The last two inequalities are contradictory, which completes the proof.

## 4 Numerical experiments

This section demonstrates the numerical efficiency of the to proposed algorithms through comparison to two existing algorithms, namely Algorithm 2.1a [49] and Rana [50]. The comparisons are based on the number of iterations (NI), the number of function evaluations (NF), and the CPU time. The parameter values for the proposed algorithms are set as *ρ* = 0.9, *σ* = 10^−4^, while the parameter values for Algorithm 2.1a are adopted from [49] and the parameters for Rana method are also adopted from [50]. Each method is implemented in MATLAB R2023a and executed on a PC equipped with an Infinix laptop featuring an Intel Core i7 processor, 32.0 GB of RAM, and a high-performance configuration. The termination criteria for the four algorithms are set as ‖*F*_*k*_‖ ≤ 10^−5^.

The benchmark problems considered for the experiments are presented below with dimensions of *n* = 1000;5, 000;10, 000;50, 000;100, 000, and five different initial points: *x*_1_ = (1, 1, 1,…, 1)^*T*^, *x*_2_ = (0.6, 0.6,…, 0.6)^*T*^, *x*_3_ = (0.5, 0.5,…, 0.5)^*T*^, *x*_4_(0.4, 0.4,…, 0.4)^*T*^, *x*_5_(0.1, 0.1,…, 0.1)^*T*^.

**Problem 4.1** ([7]). Exponential Function:

$$\begin{array}{c} f_{1}=e^{x_{1}}-1 \end{array}$$

$$\begin{array}{c} f_{i}=e^{x_{i}}+x_{i}-1,\;\;i=2,3,\cdots ,n,\Omega =R_{+}^{n} \end{array}$$

**Problem 4.2** ([8]). Logarithmic Function:

$$\begin{array}{c} f_{i}\left(x\right)=2x_{i}-\text{sin}\left|x_{i}\right|,\;\text{for}\;i=1,2,3,\ldots ,n. \end{array}$$

$$\begin{array}{c} \Omega =x\in R:\sum_{i=1}^{n}x_{i}\leq n,\;x_{i}\geq 0,\;\text{for}\;i=1,2,\ldots ,n. \end{array}$$

**Problem 4.3** ([9]). Modified Semismooth function:

$$\begin{array}{c} f_{i}\left(x\right)=cos\left(x_{i}\right)+\left(x_{i}\right)-1; \end{array}$$

**Problem 4.4** ([7]). Tridiagonal exponential function:

$$\begin{array}{c} f_{1}\left(x\right)=x_{1}-e^{cos(h(x_{1}+x_{2}))}, \end{array}$$

$$\begin{array}{c} f_{i}\left(x\right)=x_{i}-e^{cos(h(x_{i-1}+x_{i}+x_{i+1}))},\;i=2,\cdots ,n-1, \end{array}$$

$$\begin{array}{c} f_{n}\left(x\right)=x_{n}-e^{cos(h(x_{n-1}+x_{n}))}. \end{array}$$

$$\begin{array}{c} h=\frac{1}{n+1},\;\;\Omega =R_{+}^{n} \end{array}$$

**Problem 4.5** ([8]). Nonsmooth Function

$$\begin{array}{c} f_{i}\left(x\right)=x_{i}-\text{sin}\left|x_{i}-1\right|,\;\text{for}\;i=1,2,3,\ldots ,n. \end{array}$$

$$\begin{array}{c} \Omega =x\in R:\sum_{i=1}^{n}x_{i}\leq n,\;x_{i}\geq -1,\;\text{for}\;i=1,2,\ldots ,n. \end{array}$$

**Problem 4.6** ([11]). Modified exponential Function:

$$\begin{array}{c} f_{i}={\left(e^{x_{i}}\right)}^{2}+\frac{1}{2}sin\left(2x_{i}\right)-1,\;\;i=2,3,\cdots ,n,\;\;\Omega =R_{+}^{n} \end{array}$$

The computational results obtained from the experiments are presented in the Tables 1–6 below.

**Table 1:** Comparison of numerical performance metrics for SRMIL, SMRMIL, Algorithm 2.1a, and Rana on Problem 4.1.

DIM	Initial Point	SRMIL			SMRMIL			Algorithm 2.1a			Rana		
		Iter	Fval	CPU	Iter	Fval	CPU	Iter	Fval	CPU	Iter	Fval	CPU
1000	x_1	6	77	0.021835	6	77	0.027228	9	20	0.012597	***	***	***
	x_2	6	74	0.013992	6	74	0.016784	8	18	0.00848	7	80	0.013602
	x_3	6	74	0.009607	6	74	0.007175	8	18	0.006545	***	***	***
	x_4	5	63	0.007566	5	63	0.008879	8	18	0.003495	8	97	0.011173
	x_5	6	72	0.012442	6	72	0.0156	8	18	0.009182	6	64	0.013272
5000	x_1	6	75	0.035139	6	75	0.032401	10	22	0.017832	8	97	0.03707
	x_2	6	73	0.029741	6	73	0.023001	9	20	0.010769	8	81	0.03141
	x_3	6	76	0.028753	6	76	0.027113	9	20	0.01082	7	78	0.029569
	x_4	7	86	0.030097	7	86	0.030201	9	20	0.011249	***	***	***
	x_5	5	62	0.02252	5	62	0.017023	8	18	0.006302	***	***	***
10000	x_1	6	75	0.040982	6	75	0.041572	10	22	0.015938	5	56	0.029637
	x_2	6	73	0.047855	6	73	0.038099	9	20	0.01812	29	329	0.164415
	x_3	5	61	0.034187	5	63	0.033664	9	20	0.016712	6	54	0.028129
	x_4	7	85	0.056811	7	85	0.042874	9	20	0.014729	***	***	***
	x_5	4	49	0.02807	4	49	0.026671	8	18	0.013208	8	96	0.048363
50000	x_1	6	74	0.212681	6	74	0.142194	10	22	0.092565	5	55	0.121906
	x_2	6	73	0.200917	6	73	0.159506	9	20	0.062572	11	123	0.262899
	x_3	4	50	0.130826	4	50	0.108853	9	20	0.052273	7	86	0.170084
	x_4	7	84	0.22147	7	84	0.182095	9	20	0.054119	20	198	0.483241
	x_5	7	84	0.194464	7	84	0.185748	9	20	0.053481	33	342	0.721498
100000	x_1	6	74	0.395316	6	74	0.289376	11	24	0.14752	5	50	0.222027
	x_2	6	73	0.318569	6	73	0.302256	9	20	0.11479	10	113	0.537027
	x_3	6	76	0.3365	6	76	0.305745	10	22	0.12007	***	***	***
	x_4	7	84	0.369268	7	84	0.345065	10	22	0.12942	5	48	0.201215
	x_5	7	83	0.34923	7	83	0.353205	9	20	0.10331	5	51	0.214722

**Table 2:** Comparison of numerical performance metrics for SRMIL, SMRMIL, Algorithm 2.1a, and Rana on Problem 4.2.

DIM	Initial Point	SRMIL			SMRMIL			Algorithm 2.1a			Rana		
		Iter	Fval	CPU	Iter	Fval	CPU	Iter	Fval	CPU	Iter	Fval	CPU
1000	x_1	8	25	0.005438	9	28	0.005391	28	58	0.010177	3	11	0.003976
	x_2	8	25	0.002955	8	24	0.003318	28	58	0.007759	7	26	0.004728
	x_3	8	24	0.003752	8	25	0.002704	27	56	0.008787	5	18	0.00343
	x_4	8	24	0.003089	8	25	0.003548	27	56	0.007968	7	28	0.005145
	x_5	7	21	0.003116	8	24	0.003521	24	50	0.006505	7	28	0.003818
5000	x_1	9	29	0.011972	8	25	0.010084	30	62	0.0228	6	23	0.014888
	x_2	8	24	0.011095	9	28	0.012698	29	60	0.021074	7	28	0.012942
	x_3	8	24	0.012237	8	24	0.010097	29	60	0.025594	7	28	0.01226
	x_4	8	24	0.009013	8	24	0.012682	29	60	0.023501	7	28	0.010345
	x_5	8	25	0.012633	8	24	0.012316	26	54	0.023904	7	28	0.010278
10000	x_1	9	28	0.018461	9	28	0.018695	31	64	0.04398	3	11	0.010232
	x_2	7	21	0.012915	8	24	0.015615	30	62	0.042026	7	26	0.021034
	x_3	8	24	0.016801	9	27	0.021789	30	62	0.041444	7	28	0.018846
	x_4	9	28	0.017869	9	27	0.017784	29	60	0.043033	7	28	0.022013
	x_5	7	21	0.01488	8	24	0.015426	27	56	0.04421	3	10	0.010379
50000	x_1	8	25	0.061795	8	25	0.06216	32	66	0.1732	8	33	0.102152
	x_2	9	28	0.080082	9	28	0.077281	32	66	0.17761	5	18	0.071975
	x_3	7	21	0.060066	7	21	0.05205	31	64	0.16376	8	32	0.089239
	x_4	9	28	0.065815	9	28	0.063228	31	64	0.16549	8	32	0.097812
	x_5	9	27	0.061734	8	24	0.065054	28	58	0.14916	7	28	0.090329
100000	x_1	8	25	0.131644	10	31	0.153626	33	68	0.35237	8	33	0.193181
	x_2	7	21	0.096228	7	21	0.105231	32	66	0.37302	8	32	0.194552
	x_3	7	21	0.101898	7	21	0.108397	32	66	0.34805	8	32	0.186402
	x_4	7	21	0.102652	7	21	0.121734	31	64	0.34549	8	32	0.195935
	x_5	7	21	0.110887	7	21	0.097865	29	60	0.31615	7	28	0.16842

**Table 3:** Comparison of numerical performance metrics for SRMIL, SMRMIL, Algorithm 2.1a, and Rana on Problem 4.3.

DIM	Initial Point	SRMIL			SMRMIL			Algorithm 2.1a			Rana		
		Iter	Fval	CPU	Iter	Fval	CPU	Iter	Fval	CPU	Iter	Fval	CPU
1000	x_1	5	11	0.002755	5	11	0.003316	22	46	0.006997	3	7	0.004368
	x_2	5	11	0.001836	5	11	0.002835	23	48	0.005875	8	30	0.00392
	x_3	4	9	0.002623	4	9	0.001894	21	44	0.006623	7	26	0.005534
	x_4	4	9	0.00234	4	9	0.001748	20	42	0.006401	2	5	0.002458
	x_5	3	7	0.002472	3	7	0.001756	19	40	0.005798	6	23	0.004497
5000	x_1	5	11	0.005497	5	11	0.005175	23	48	0.024349	8	28	0.011681
	x_2	5	11	0.004712	5	11	0.006459	24	50	0.017579	8	30	0.013958
	x_3	4	9	0.005327	4	9	0.004897	23	48	0.020059	8	30	0.012075
	x_4	4	9	0.006509	4	9	0.004779	21	44	0.024879	7	26	0.009757
	x_5	3	7	0.003989	3	7	0.003993	21	44	0.01785	3	9	0.007931
10000	x_1	5	11	0.010032	5	11	0.008662	24	50	0.03448	4	10	0.010665
	x_2	5	11	0.010469	5	11	0.009491	25	52	0.038413	8	30	0.022286
	x_3	4	9	0.007379	4	9	0.007544	23	48	0.02997	3	8	0.008566
	x_4	4	9	0.006477	4	9	0.008741	22	46	0.033447	7	26	0.017928
	x_5	3	7	0.005391	3	7	0.005141	22	46	0.031081	7	27	0.017987
50000	x_1	5	11	0.032206	5	11	0.029736	25	52	0.13667	8	28	0.083158
	x_2	5	11	0.033498	5	11	0.035728	26	54	0.15246	9	34	0.083107
	x_3	4	9	0.023338	4	9	0.024671	25	52	0.13214	8	30	0.089134
	x_4	4	9	0.024794	4	9	0.024077	23	48	0.12266	7	26	0.0794
	x_5	3	7	0.021582	3	7	0.021611	23	48	0.11803	7	27	0.068833
100000	x_1	5	11	0.056254	5	11	0.061971	26	54	0.29351	9	32	0.200293
	x_2	6	14	0.067883	6	14	0.078676	27	56	0.31751	9	34	0.209383
	x_3	5	12	0.062394	5	12	0.064767	26	54	0.30076	8	30	0.162584
	x_4	4	9	0.051783	4	9	0.048158	24	50	0.28348	7	26	0.141119
	x_5	3	7	0.041679	3	7	0.036234	24	50	0.26721	7	27	0.135787

**Table 4:** Comparison of numerical performance metrics for SRMIL, SMRMIL, Algorithm 2.1a, and Rana on Problem 4.4.

DIM	Initial Point	SRMIL			SMRMIL			Algorithm 2.1a			Rana		
		Iter	Fval	CPU	Iter	Fval	CPU	Iter	Fval	CPU	Iter	Fval	CPU
1000	x_1	41	353	0.0564	39	352	0.045502	30	62	0.01386	104	817	0.132278
	x_2	42	364	0.043118	39	352	0.039421	30	62	0.015153	49	385	0.056053
	x_3	42	364	0.046676	40	365	0.053566	30	62	0.012951	84	657	0.081507
	x_4	42	364	0.061407	40	365	0.043197	30	62	0.016055	84	672	0.08249
	x_5	40	364	0.046761	42	365	0.050236	31	64	0.012764	87	684	0.080639
5000	x_1	39	331	0.169155	40	339	0.163547	31	64	0.044333	72	573	0.270583
	x_2	40	338	0.166177	40	339	0.154065	32	66	0.040826	97	760	0.35078
	x_3	40	338	0.149384	40	339	0.151027	32	66	0.037902	97	760	0.337734
	x_4	40	338	0.15945	40	339	0.157961	32	66	0.040835	70	541	0.250202
	x_5	40	338	0.15449	40	339	0.154522	32	66	0.044203	68	542	0.242662
10000	x_1	38	321	0.29772	38	320	0.287728	32	66	0.076571	90	701	0.611857
	x_2	39	332	0.285995	39	331	0.279505	33	68	0.073652	91	704	0.603588
	x_3	39	332	0.285528	39	330	0.291667	33	68	0.075859	91	704	0.604748
	x_4	39	332	0.313041	39	330	0.28254	33	68	0.07865	91	704	0.615823
	x_5	39	332	0.287036	37	327	0.278907	33	68	0.067394	89	688	0.630287
50000	x_1	36	298	1.258429	34	295	1.093561	34	70	0.33286	84	643	2.436733
	x_2	37	305	1.264824	35	301	1.075918	34	70	0.34257	84	643	2.445876
	x_3	37	305	1.158613	35	301	1.097695	34	70	0.33152	84	643	2.44862
	x_4	37	305	1.181369	35	301	1.146183	34	70	0.3356	84	643	2.413309
	x_5	37	305	1.153495	35	301	1.203868	35	72	0.31767	84	643	2.417932
100000	x_1	36	295	2.454618	36	291	2.738724	34	70	0.69199	55	421	3.431026
	x_2	36	294	2.43618	35	299	3.015436	35	72	0.76991	46	342	2.804873
	x_3	36	293	2.402104	35	299	2.753881	35	72	0.73557	74	564	4.649494
	x_4	36	293	2.417126	35	299	2.674906	35	72	0.7062	72	548	4.605459
	x_5	37	303	2.492532	35	299	2.42305	35	72	0.72616	68	518	4.264912

**Table 5:** Comparison of numerical performance metrics for SRMIL, SMRMIL, Algorithm 2.1a, and Rana on Problem 4.5.

DIM	Initial Point	SRMIL			SMRMIL			Algorithm 2.1a			Rana		
		Iter	Fval	CPU	Iter	Fval	CPU	Iter	Fval	CPU	Iter	Fval	CPU
1000	x_1	4	34	0.004902	4	34	0.004616	10	22	0.004482	2	12	0.003181
	x_2	4	34	0.006054	4	34	0.003173	9	20	0.002621	3	22	0.003388
	x_3	3	25	0.002928	3	25	0.002918	8	18	0.003223	2	11	0.002316
	x_4	4	33	0.003916	4	33	0.003485	9	20	0.003211	3	21	0.003472
	x_5	5	41	0.003852	5	41	0.005687	10	22	0.004283	3	21	0.003935
5000	x_1	4	34	0.01179	4	34	0.013682	11	24	0.011861	2	12	0.007594
	x_2	5	42	0.013845	5	42	0.015328	10	22	0.009713	3	22	0.012853
	x_3	4	33	0.013021	4	33	0.012009	8	18	0.007726	2	11	0.007961
	x_4	4	33	0.01449	4	33	0.012755	10	22	0.010475	3	21	0.014278
	x_5	5	41	0.014578	5	41	0.012433	11	24	0.009803	4	31	0.017096
10000	x_1	5	42	0.023198	5	42	0.024201	11	24	0.017849	4	32	0.020211
	x_2	5	42	0.026479	5	42	0.022338	10	22	0.016463	2	12	0.009672
	x_3	4	33	0.018669	4	33	0.019347	8	18	0.013132	2	11	0.011325
	x_4	4	33	0.020122	4	33	0.01975	10	22	0.015952	3	21	0.015057
	x_5	5	41	0.02131	5	41	0.021186	11	24	0.016958	4	31	0.018565
50000	x_1	5	42	0.095127	5	42	0.089027	12	26	0.067568	4	32	0.076473
	x_2	5	42	0.085896	5	42	0.086173	11	24	0.066146	4	32	0.083799
	x_3	4	33	0.075921	4	33	0.07311	9	20	0.052727	3	21	0.060576
	x_4	4	33	0.068379	4	33	0.065084	10	22	0.055427	3	21	0.061339
	x_5	5	41	0.08354	5	41	0.088197	12	26	0.066027	4	31	0.076213
100000	x_1	5	42	0.188666	5	42	0.16359	12	26	0.14356	4	32	0.163744
	x_2	5	42	0.194865	5	42	0.185779	11	24	0.13093	4	32	0.164316
	x_3	4	33	0.139817	4	33	0.140357	9	20	0.11406	3	21	0.113697
	x_4	5	41	0.192967	5	41	0.198211	11	24	0.11936	3	21	0.106315
	x_5	5	41	0.165541	5	41	0.183003	12	26	0.14623	4	31	0.158503

**Table 6:** Comparison of numerical performance metrics for SRMIL, SMRMIL, Algorithm 2.1a, and Rana on Problem 4.6.

DIM	Initial Point	SRMIL			SMRMIL			Algorithm 2.1a			Rana		
		Iter	Fval	CPU	Iter	Fval	CPU	Iter	Fval	CPU	Iter	Fval	CPU
1000	x_1	1	5	0.001966	1	5	0.0014	1	3	0.003246	1	5	0.001653
	x_2	1	4	0.001319	1	4	0.001339	8	18	0.003816	1	4	0.000982
	x_3	1	3	0.001129	1	3	0.001371	1	3	0.001156	1	3	0.001193
	x_4	1	14	0.002211	1	14	0.002118	9	20	0.00361	1	14	0.002135
	x_5	1	14	0.002158	1	14	0.002187	8	18	0.003791	1	14	0.002043
5000	x_1	1	5	0.003016	1	5	0.003114	1	3	0.001933	1	5	0.003022
	x_2	1	4	0.002553	1	4	0.002478	8	18	0.010534	1	4	0.002487
	x_3	1	3	0.002936	1	3	0.001616	1	3	0.001848	1	3	0.002205
	x_4	1	14	0.007184	1	14	0.006543	9	20	0.011774	1	14	0.007923
	x_5	1	14	0.005752	1	14	0.005079	9	20	0.011394	1	14	0.005901
10000	x_1	1	5	0.004729	1	5	0.005034	1	3	0.004583	1	5	0.005112
	x_2	1	4	0.004208	1	4	0.004585	8	18	0.018602	1	4	0.003779
	x_3	1	3	0.003307	1	3	0.003135	1	3	0.002703	1	3	0.003107
	x_4	1	14	0.012342	1	14	0.010902	9	20	0.018658	1	14	0.011917
	x_5	1	14	0.011046	1	14	0.008543	9	20	0.017224	1	14	0.009057
50000	x_1	1	5	0.018545	1	5	0.017594	1	3	0.011042	1	5	0.017512
	x_2	1	4	0.017704	1	4	0.01553	9	20	0.07737	1	4	0.012999
	x_3	1	3	0.011117	1	3	0.011617	1	3	0.009011	1	3	0.00895
	x_4	1	14	0.043272	1	14	0.03742	10	22	0.08424	1	14	0.041666
	x_5	1	14	0.035507	1	14	0.03276	9	20	0.071748	1	14	0.035934
100000	x_1	1	5	0.034756	1	5	0.032918	1	3	0.020087	1	5	0.032997
	x_2	1	4	0.026191	1	4	0.026783	9	20	0.14444	1	4	0.02698
	x_3	1	3	0.020079	1	3	0.02055	1	3	0.016248	1	3	0.018365
	x_4	1	14	0.091189	1	14	0.079832	10	22	0.16382	1	14	0.082043
	x_5	1	14	0.077883	1	14	0.063942	10	22	0.15685	1	14	0.070348

Results from Tables 1–6 show that the proposed SRMIL and SMRMIL algorithms exhibit nearly identical values for Iter, Fval, and CPU time across the tested problems. Moreover, the findings indicate that the four algorithms applied to Problems 4.1–4.6 are largely insensitive to variations in initial points and problem dimensions.

Additionally, the study utilize the performance profiles tool introduced by Dolan and Moré [51], which provide valuable insights into the efficiency and robustness of the algorithms. The performance profiles are cumulative distribution functions that measure the proportion of problems solved by an algorithm within a given performance ratio threshold. Particularly, for each method, the performance ratio is computed as the metric value (e.g., Iter, Fval, or CPU time) attained by the method on a given function divided by the best metric value attained by any method for that problem. A smaller ratio indicates better performance as illustrated in the following figures.

**Fig 1:**
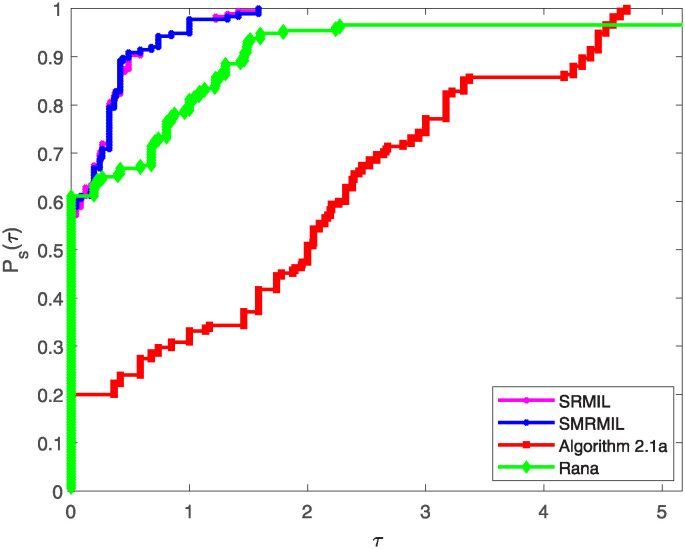
Performance profile of the four algorithms based on number of iteration.

**Fig 2:**
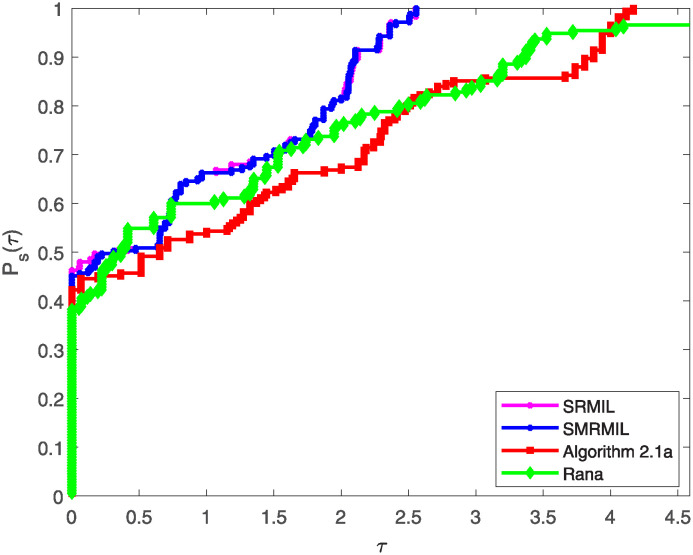
Performance profile of the four algorithms based on the function value of iteration number.

**Fig 3:**
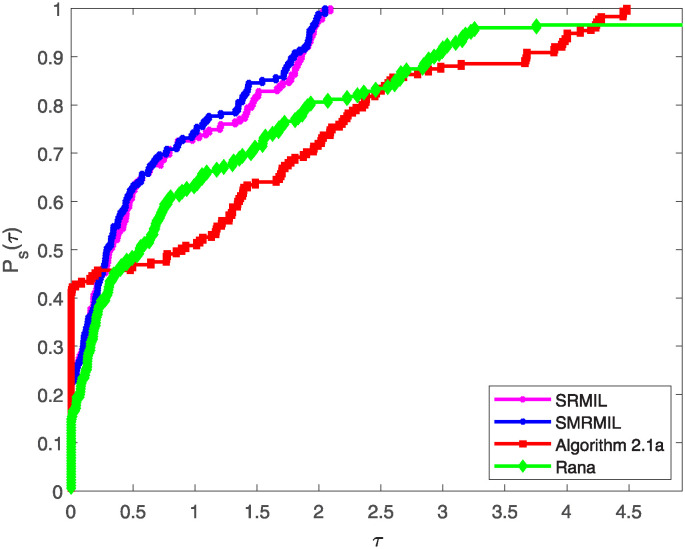
Performance profile of the four algorithms based on CPU time.

Figs 1–3 illustrates these profiles for the three evaluation criteria:

- Fig 1 represents the performance profile with respect to the number of iterations, highlighting how fast an algorithm converges to a solution.
- In Fig 2, the performance profile plot the curve for the number of function evaluations, assessing the algorithms’ efficiency in terms of computational resources utilized during the iteration process.
- Plot from Fig 3 illustrates the performance profile based on CPU time, showcasing the computational efficiency of the each algorithm.

In all three figures, SRMIL and SMRMIL which exhibit nearly identical values, consistently attain higher cumulative probabilities at various performance thresholds, indicating their superior competitiveness and efficiency compared to Algorithm 2.1a and Rana. This dominance across all performance criteria demonstrates the efficiency and practical advantage of the proposed methods.

## 5 Application

Robotic systems are increasingly becoming an integral part of modern technology, finding applications in industries ranging from manufacturing to healthcare. The three degrees of freedom (3DOF) arm robot model represents a class of manipulators characterized by its versatility and utility in complex tasks consisting of three rotational joints, each providing a degree of freedom [52]. This configuration allows the robot to perform an extensive array of movements and tasks, making it highly adaptable to various operational environments [53–55]. The kinematic equations of the 3DOF arm robot describe the position and orientation of the end-effector as functions of the joint angles while the dynamic equations govern the torques and forces required to achieve desired movements [56].

In this study, we are more interested in the discrete kinematic model equation with three degrees of freedom define below which also represents the planar three-joint kinematic model:

$$\begin{array}{c} r\left(\theta \right)=[\begin{array}{c} l_{1}\text{cos}(\theta_{1})+l_{2}\text{cos}(\theta_{1}+\theta_{2})+l_{3}\text{cos}(\theta_{1}+\theta_{2}+\theta_{3})\\ l_{1}\text{sin}(\theta_{1})+l_{2}\text{sin}(\theta_{1}+\theta_{2})+l_{3}\text{sin}(\theta_{1}+\theta_{2}+\theta_{3}) \end{array}] \end{array}$$ (60)

where *r*(⋅) denotes the kinematic transformation and positioning of a robot’s endpoint or any component relative to active adjustments in its joints, represented by $\theta \in R^{3}$. The link is denoted by *l*_*i*_ for *i* = 1, 2, 3 represents the length of the corresponding link. In general, in the context of robotic motion, *r*(*θ*) represents the position and orientation of the robot end effector, while in this specific study case it only represents the *x* − *y* cartesian robot position. Effective control and trajectory planning for such robots necessitate advanced optimization techniques to handle the non-linearity and high dimensionality of the system dynamics [53–55].

Consider $\psi_{t_{k}}\in R^{2}$ to denote the vector representing the desired path at a given time instant *t*_*k*_. We propose the following least-squares model, which will be evaluated at each time segment *t*_*k*_ within the interval [0, *t*_*f*_]. The optimization problem is formulated as follows:

$$\begin{array}{c} \underset{\theta \in R^{3}}{\text{min}}\frac{1}{2}\sum_{i=1}^{3}{\left(r_{i}\left(\theta \right)-\psi_{t_{k},i}^{\left(n\right)}\right)}^{2}, \end{array}$$ (61)

where $\psi_{t_{k},i}^{\left(n\right)}$ denotes the target path at the moment *t*_*k*_ of a *Lissajous curve*. Furthermore, various *Lissajous curves* in 3DOF have been employed, for example, see [37, 57].

To simulate the results, the pseudo-code below was used to obtain the solution of the inverse kinematic problem.

**Algorithm 2:** Solution of 3DOF robotic arm model using (SRMILCG)

**Step 1**: Inputs: Initialize parameters *t*_0_, $\theta_{t_{0}}$, *t*_*f*_, *g*, and *K*_max_

**Step 2**: For *k* = 1 to *K*_max_ do

*t*_*k*_ = k*g;

**Step 3**: Evaluate Lissajous curves $\psi_{t_{k},i}^{\left(n\right)}$;

**Step 4**: Compute $\theta_{t_{k}}$ using SRMILCG$\left(\theta_{t_{0}},\psi_{t_{k},i}^{\left(n\right)}\right)$ stated in Algorithm 1;

**Step5**: Set $\theta_{\text{new}}=[\theta_{t_{0}};\theta_{t_{k}}]$;

**Step 6**: Output: *θ*_new_

**Remark 5.1.** When applying the CG methods to the specific case of the 3DOF robotic arm, the continuity of the model naturally follows from the well-defined kinematic equations of the robotic system. While monotonicity is not explicitly proven for the specific 3DOF case, the embedding of the kinematic equations into the optimization problem aligns well with the assumptions of the CG framework.

To solve the model and then simulate the results, the following parameters are used in the implementation:

- At the initial time instant *t*_0_ = 0, the joint angular vector is $\theta_{t_{0}}=[0,\frac{\pi }{3},\frac{\pi }{2}]$,
- The link has a length of *l*_*i*_ (where *i* = 1, 2, 3),
- The anticipated total task duration is *t*_*f*_ = 10 *seconds*.

The algorithms used in this experiment were developed using MATLAB R2022a, and the computations were performed on an Intel (R) CORE(TM) i7-3537U processor with a clock speed of 2.00 GHz and 8 GB of RAM. The effectiveness of the new formulae SRMIL and SMRMIL is demonstrated by comparing their results with those of other existing algorithms that share similar characteristics. For this comparison in numerical experiments, we consider the following methods:

1. The Rivaie- Mustafa-Ismail-Leong (RMIL) CG method by [25] with the following parameter: $\beta_{k}^{RMIL}=\frac{F_{k+1}^{T}y_{k}}{\left|\right|d_{k}{\left|\right|}^{2}}.$
2. The modified RMIL method (MRMIL) by [26] given as: $\beta_{k}^{MRMIL}=\frac{F_{k+1}^{T}\left(F_{k+1}-F_{k}-d_{k}\right)}{\left|\right|d_{k}{\left|\right|}^{2}}.$
3. The CG_Descent method by Hager and Zhang [58], defined by the following formula: $\beta_{k}^{N}=\frac{1}{d_{k}^{T}y_{K}}{\left(y_{k}-2d_{k}\frac{∥y_{k}{∥}^{2}}{d_{k}^{T}y_{k}}\right)}^{T}F_{k+1}$.

**Problem 5.1**. Consider the following Lissajous curve presented in [59, 60] defined by:

$$\begin{array}{c} \psi_{t_{k}}^{\left(1\right)}=[\begin{array}{c} 1.5+0.4\text{sin}(\frac{\pi t_{k}}{5})\\ \frac{\sqrt{3}}{2}+0.4\text{sin}\left(\frac{\pi t_{k}}{5}+\frac{\pi }{3}\right) \end{array}]. \end{array}$$ (62)

**Fig 4:**
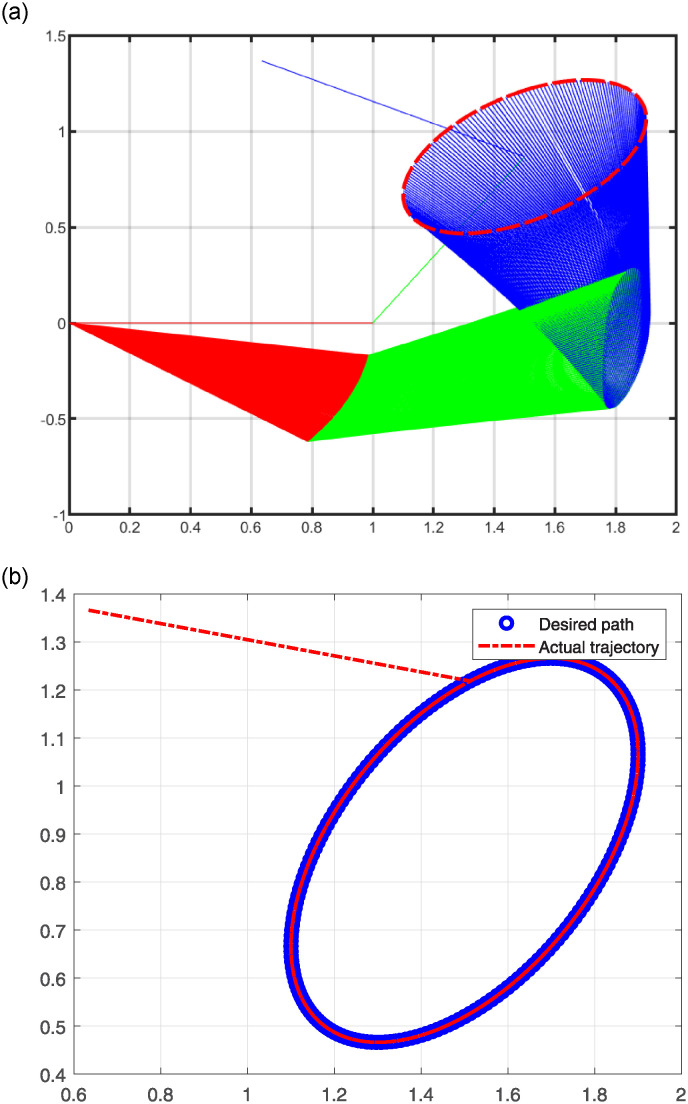
Generated robot trajectories following the Lissajous curve (a); End-effector path and intended path of Lissajous curve (b).

**Fig 5:**
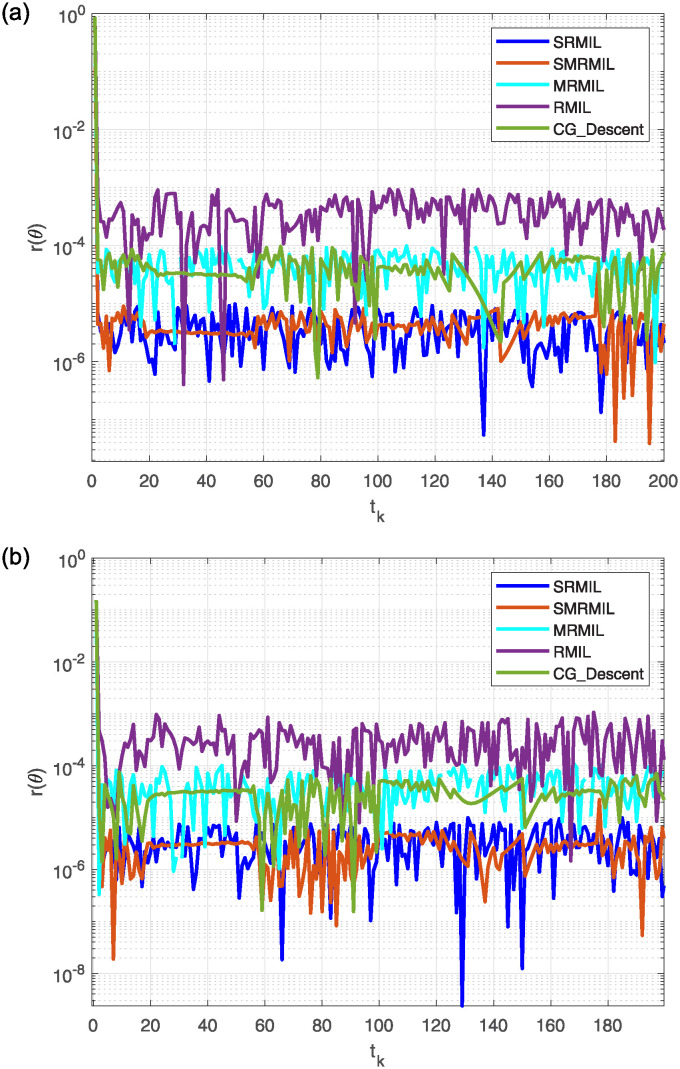
Observing the Lissajous curve’s residual error: Along *x* axis (a), along *y* axis (b).

The numerical results are illustrated in the following figures. In particular, Fig 4(a) depicts the robot trajectories generated by the Algorithm 2. Fig 4(b) effectively illustrates the successful synthesis of robot trajectories for the task given. Moreover, Fig 5(a) and 5(b) display the residual error for each algorithm used in the experiment.

After analyzing the Figures that show the output of solving Eq (60) with the corresponding Lissajous curve (62) using the proposed and compared algorithms, Fig 4(a) shows the robot’s end effector model precisely following the target path. The success of creating robot trajectories for the assignment is aptly illustrated in Fig 4(b). The residual error rates are shown in Fig 5(a) and 5(b), respectively. SRMIL and SMRMIL have the lowest error at about 10^−6^, followed by CG_Descent at 10^−5^, MRMIL at 10^−4^, and RMIL at 10^−3^. The algorithms exhibit good performance in this example, as evidenced by the impressively low residual error rates of SRMIL and SMRMIl, which confirms their efficacy in finding the accuracy on the solution of the inverse kinematic problem.

**Problem 5.2**. Consider the following Lissajous curve given in [61] defined by:

$$\begin{array}{c} \psi_{t_{k}}^{\left(2\right)}=[\begin{array}{c} \frac{3}{2}+\frac{1}{5}\text{sin}\left(t_{k}\right)\\ \frac{\sqrt{3}}{2}+\frac{1}{5}\text{sin}\left(2t_{k}\right) \end{array}], \end{array}$$ (63)

**Fig 6:**
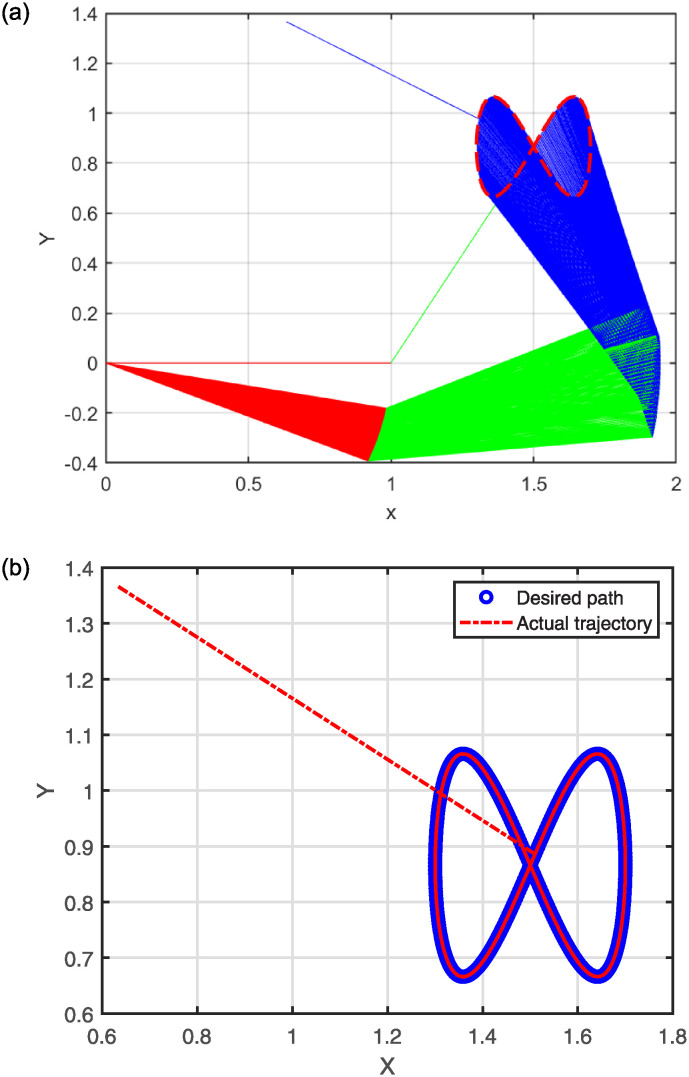
Generated robot trajectories following the Lissajous curve (a); End-effector path and intended path of Lissajous curve (b).

**Fig 7:**
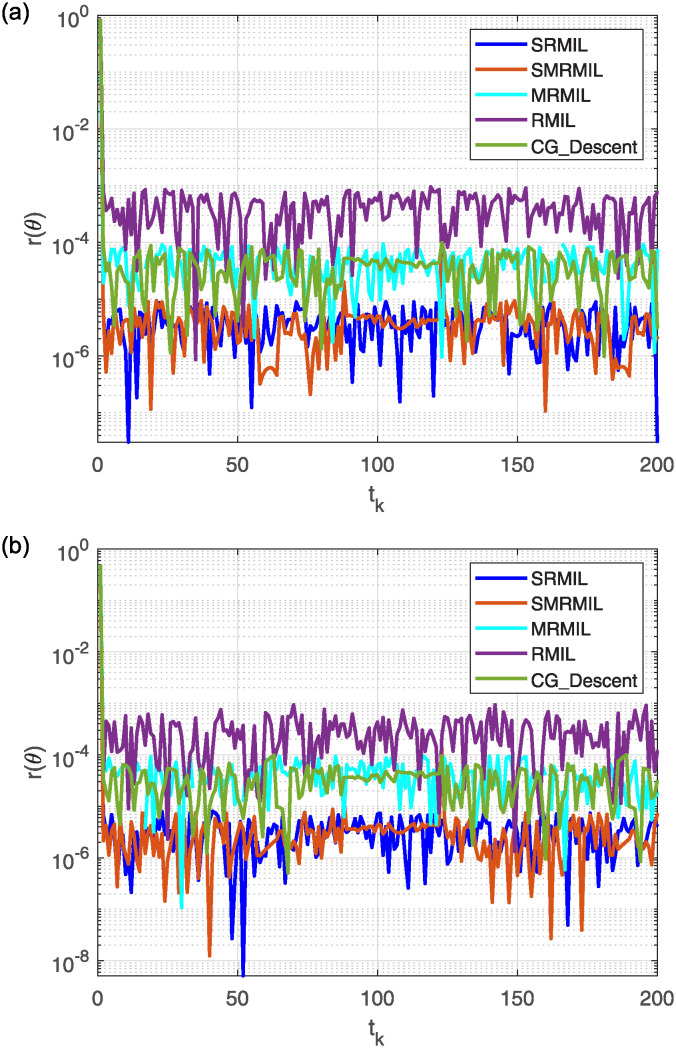
Observing the Lissajous curve’s residual error: Along *x* axis (a), along *y* axis (b).

The simulation results for problem 2 are presented in the following figures. Specifically, Fig 6(a) shows the robot trajectories generated by Algorithm 2. Fig 6(b) effectively illustrates the successful synthesis of robot trajectories for the given task. Additionally, Fig 7(a) and 7(b) display the residual error for each algorithm used in the experiment.

The plots in Fig 6 depict the output data from the model’s solution. Specifically, Fig 6(b) illustrates how the robot trajectories completed the task, with the end-effector following a precise path. Fig 7 shows the residual error norm rates, revealing that SMRMIL has the lowest error rate at approximately 10^−6^, followed by SRMIL, CG_Descent, MRMIL and RMIL, with error rates of around 10^−5^, 10^−4^, 10^−4^, and 10^−3^, respectively.

**Problem 5.3**. The end-effector is guided to follow a Lissajous curve [57], represented as

$$\begin{array}{c} \psi_{t_{k}}^{\left(3\right)}=[\begin{array}{c} 1.5+0.2\text{sin}(4t_{k})\\ \frac{\sqrt{3}}{2}+0.2\text{sin}\left(3t_{k}\right) \end{array}], \end{array}$$ (64)

**Fig 8:**
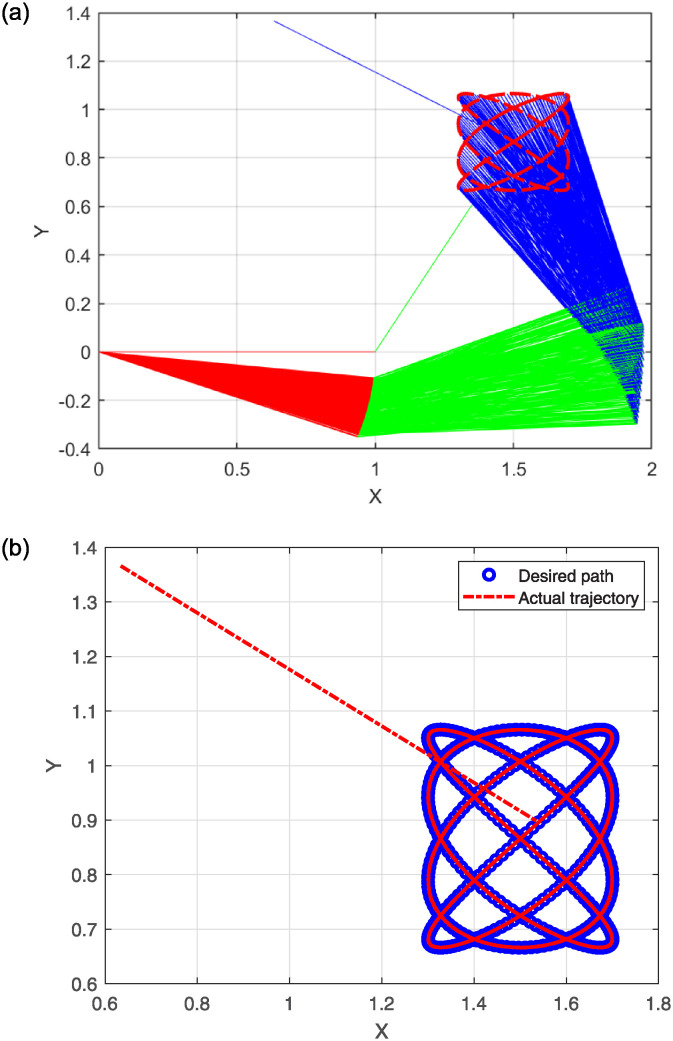
Generated robot trajectories following the Lissajous curve (a); End-effector path and intended path of Lissajous curve (b).

**Fig 9:**
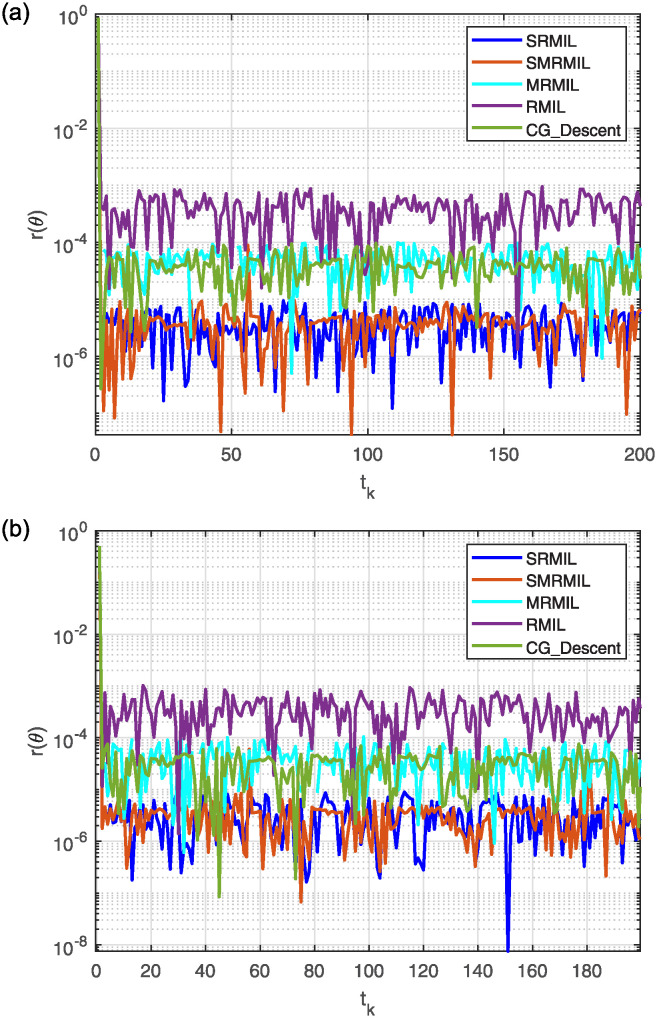
Observing the Lissajous curve’s residual error: Along *x* axis (a), along *y* axis (b).

The simulation results for problem 3 are depicted in the following figures. Fig 8(a) displays the robot trajectories produced by Algorithm 2. Fig 8(b) effectively demonstrates the successful synthesis of robot trajectories for the specified task. Moreover, Fig 9(a) and 9(b) show the residual error for each algorithm utilized in the experiment.

The plots in Fig 8 showcase the output data derived from the model’s solution. Specifically, Fig 8(b) demonstrates the robot trajectories completing the task, with the end-effector precisely following its intended path. Fig 9 presents the residual error norm rates, indicating that the SMRMIL algorithm achieves the lowest error rate at approximately 10^−7^. This is followed by the SRMIL, CG_Descent, MRMIL, and RMIL algorithms, which have error rates around 10^−6^, 10^−5^, 10^−5^, and 10^−3^, respectively. These results highlight the superior accuracy of the SMRMIL algorithm in minimizing residual error.

**Problem 5.4**. The following describes how the end-effector is guided to follow a Lissajous curve:

$$\begin{array}{c} \psi_{t_{k}}^{\left(4\right)}=[\begin{array}{c} \frac{3}{2}+\frac{1}{5}\text{cos}\left(4t_{k}\right)\\ \frac{\sqrt{3}}{2}+\frac{1}{5}\text{cos}\left(3t_{k}\right) \end{array}], \end{array}$$ (65)

**Fig 10:**
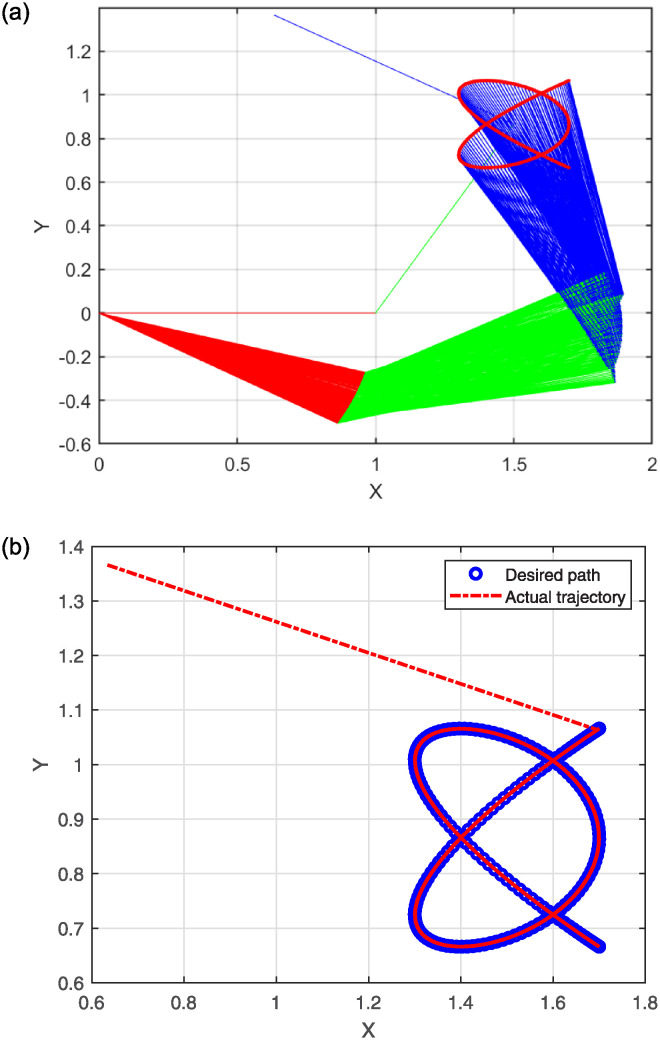
Generated robot trajectories following the Lissajous curve (a); End-effector path and intended path of Lissajous curve (b).

**Fig 11:**
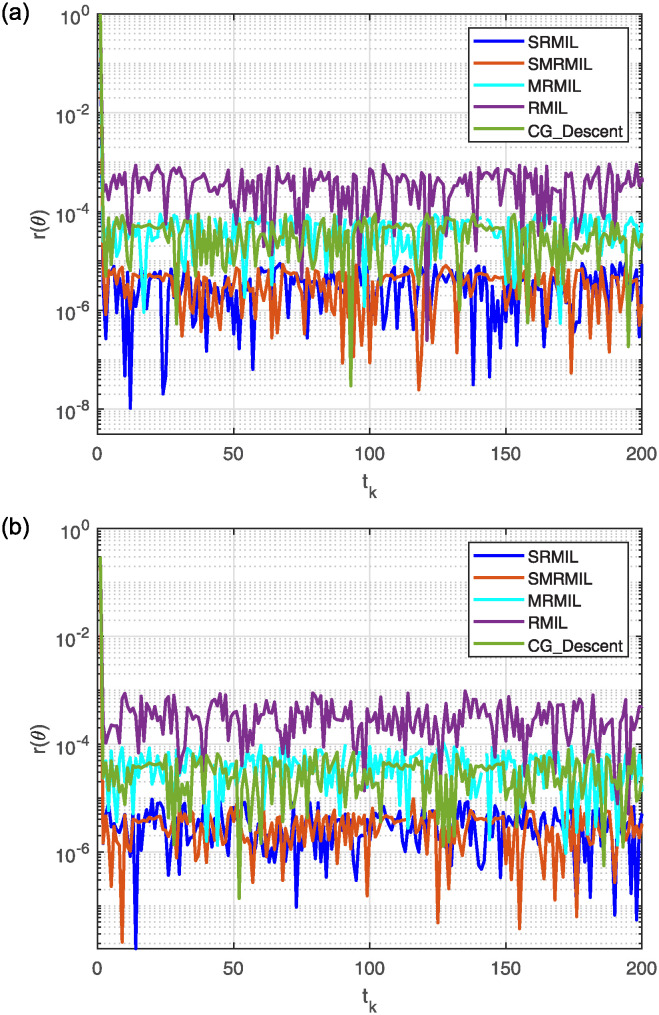
Observing the Lissajous curve’s residual error: Along *x* axis (a), along *y* axis (b).

The ensuing figures show the simulation results for problem 4. The robot trajectories generated by Algorithm 2 are shown in Fig 10(a). The synthesis of robot trajectories for the given job is successfully demonstrated in Fig 10(b). Additionally, the residual error for each method used in the experiment is displayed in Fig 11(a) and 11(b).

Fig 10 displays the plots of output data obtained from the solution of the model. In particular, Fig 10(b) illustrates the robot trajectories completing the task, with the end-effector precisely following its designed path. This indicates the robustness of the model in guiding the end-effector along the intended trajectory.

Furthermore, Fig 11 presents the residual error norm rates, providing a comparative analysis of the algorithms’ performance. The SRMIL algorithm achieves the lowest error rate at approximately 10^−7^, showcasing its superior accuracy. Following SRMIL, the SMRMIL, CG_Descent, MRMIL, and RMIL algorithms have error rates of around 10^−6^, 10^−5^, 10^−5^, and 10^−3^, respectively. These results highlight the enhanced precision of the SRMIL algorithm in minimizing residual error compared to the other methods.

**Problem 5.5**. As shown below, the end-effector is directed to follow a Lissajous curve:

$$\begin{array}{c} \psi_{t_{k}}^{\left(5\right)}=[\begin{array}{c} \frac{3}{2}+0.3\text{sin}\left(4t_{k}+\frac{2\pi }{3}\right)\\ \frac{\sqrt{3}}{2}+0.3\text{cos}\left(3t_{k}+\frac{2\pi }{3}\right) \end{array}], \end{array}$$ (66)

**Fig 12:**
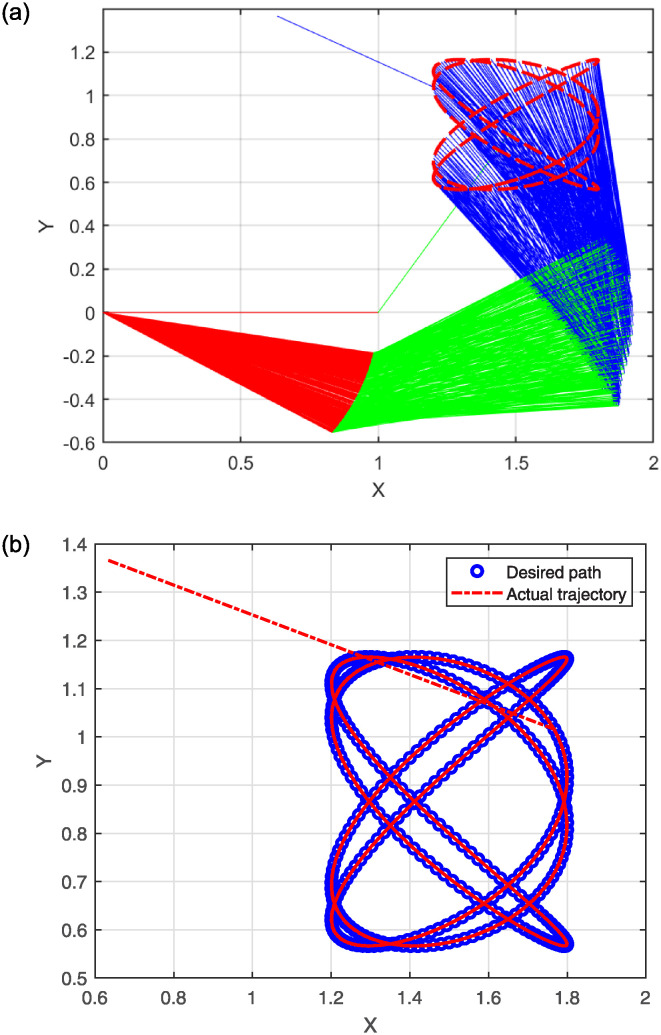
Generated robot trajectories following the Lissajous curve (a); End-effector path and intended path of Lissajous curve (b).

**Fig 13:**
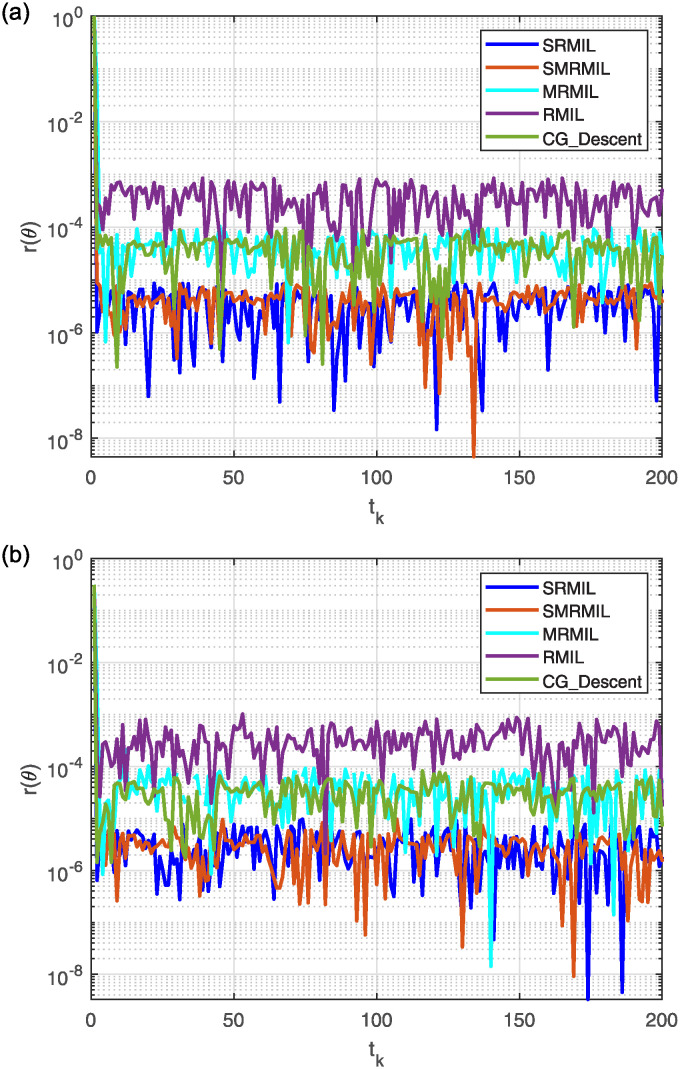
Observing the Lissajous curve’s residual error: Along *x* axis (a), along *y* axis (b).

The following figures present the simulation results for problem 5. Fig 12(a) illustrates the robot trajectories produced by Algorithm 2. Fig 12(b) demonstrates the successful synthesis of robot trajectories for the specified task. Furthermore, Fig 13 shows the residual errors for each method employed in the experiment.

Fig 13 illustrates the residual error norm rates, offering a comparative analysis of the algorithms’ performances. The SMRMIL algorithm demonstrates superior accuracy with the lowest error rate, approximately 10^−6^. It is followed by the SRMIL, CG_Descent, MRMIL, and RMIL algorithms, which exhibit error rates around 10^−6^, 10^−5^, 10^−5^, and 10^−3^, respectively. These findings underscore the enhanced precision of the SRMIL and SMRMIL algorithms in reducing residual error compared to the other methods. Furthermore, Table 7 presents the results of the problems, detailing the number of iterations, CPU time, and residual error. Regarding iteration counts, the SMRMIL method performs the best, achieving the fewest iterations, followed closely by SRMIL, CG_Descent, MRMIL, and RMIL, which have the highest count. Additionally, regarding CPU time, SMRMIL solves the problems in the shortest computational time, outperforming the SRMIL, CG_Descent, MRMIL, and RMIL methods.

**Table 7:** The performance of the SRMIL and SMRIL algorithms compared to MRMIL, RMIL, and CG_Descent algorithms on the Problems 5.1 to 5.6.

Methods	SRMIL			SMRMIL			MRMIL			RMIL			CG_Descent		
	Iter	CPU	Re	Iter	CPU	Re	Iter	CPU	Re	Iter	CPU	Re	Iter	CPU	Re
**Problem 5.1**	23	0.235	10^−6^	21	0.126	10^−6^	56	0.425	10^−4^	178	1.213	10^−3^	32	0.398	10^−5^
**Problem 5.2**	37	0.435	10^−5^	33	0.248	10^−6^	86	0.543	10^−4^	109	1.431	10^−3^	62	0.398	10^−4^
**Problem 5.3**	42	0.378	10^−6^	39	0.299	10^−7^	77	0.824	10^−5^	92	2.028	10^−3^	81	0.496	10^−5^
**Problem 5.4**	39	0.338	10^−7^	33	0.233	10^−6^	78	0.514	10^−5^	104	1.176	10^−3^	54	0.597	10^−5^
**Problem 5.5**	35	0.438	10^−6^	28	0.433	10^−6^	56	0.469	10^−5^	169	1.103	10^−3^	49	0.604	10^−5^
**Problem 5.6**	51	0.687	10^−6^	42	0.512	10^−6^	97	0.886	10^−5^	194	1.738	10^−3^	85	0.754	10^−5^

**Problem 5.6** Consider a trajectory that passes through a singularity or requires the end effector to transition between quadrants.

$$\begin{array}{c} \psi_{t_{k}}^{\left(6\right)}=[\begin{array}{c} \frac{1}{2}\text{sin}\left(t_{k}\right)\\ \frac{1}{2}\text{sin}\left(t_{k}\right)\text{cos}\left(t_{k}\right) \end{array}], \end{array}$$ (67)

This trajectory does pass through points where both components become zero at *t*_*k*_ = *nπ* (where *n* is an integer), which could indicate potential singularities in the robotic system depending on its configuration and the corresponding Jacobian.

**Fig 14:**
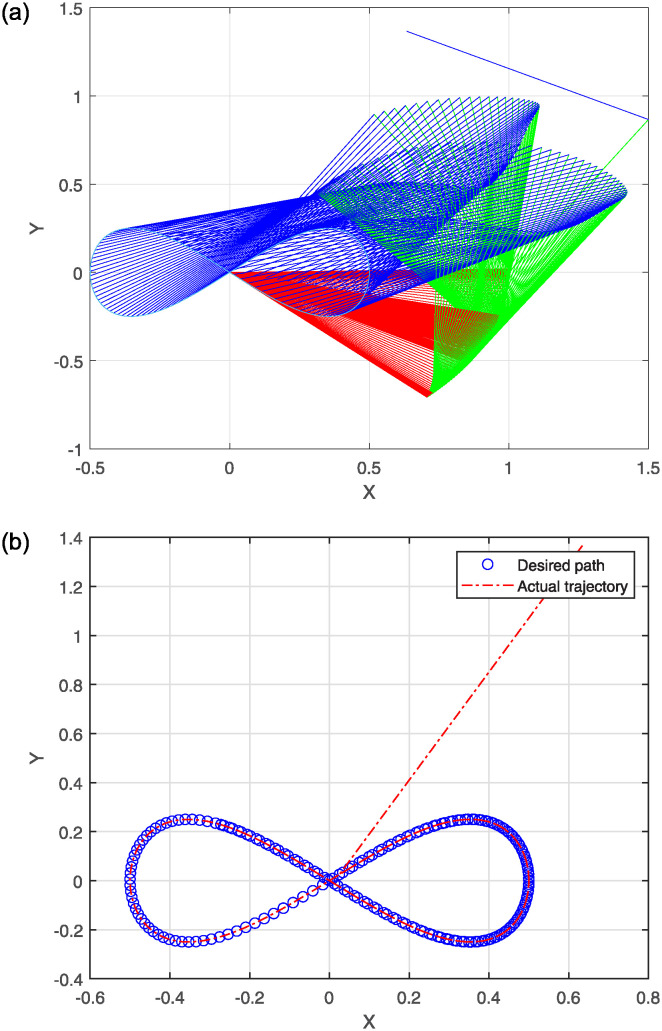
Generated robot trajectories following the Lissajous curve (a); End-effector path and intended path of Lissajous curve (b).

**Fig 15:**
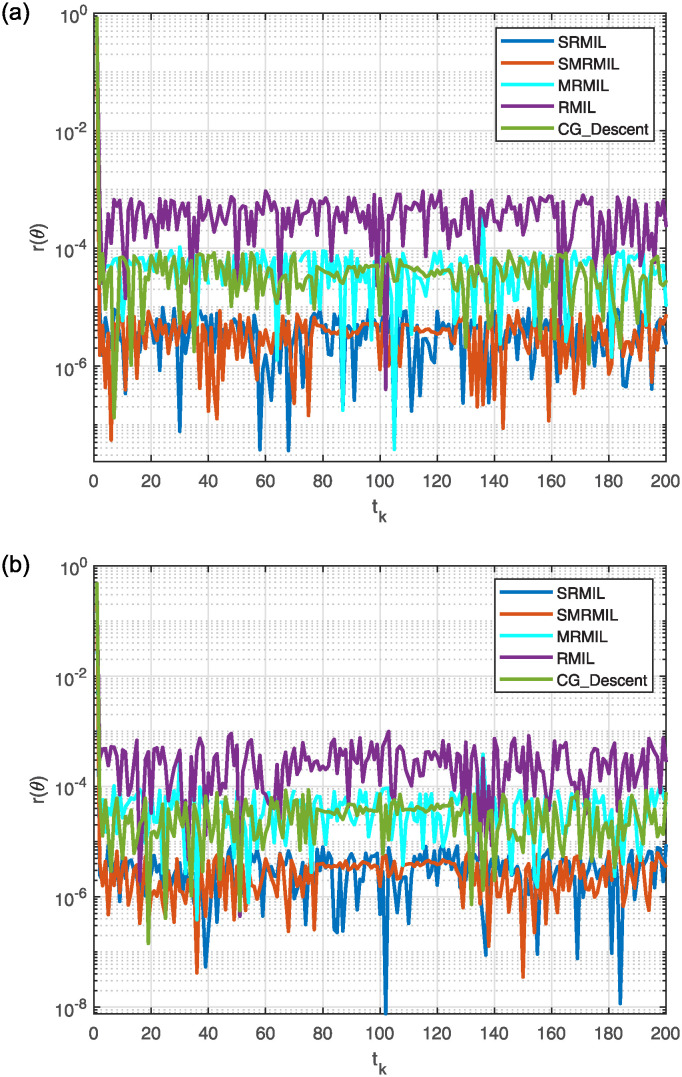
Observing the Lissajous curve’s residual error: Along *x* axis (a), along *y* axis (b).

The following figures showcase the simulation outcomes for Problem 5.6 Fig 14(a) depicts the robot trajectories generated by Algorithm 2, while Fig 14(b) highlights the successful synthesis of robot trajectories tailored to the specified task. Moreover, Fig 15 shows the residual errors for each method employed in the experiment.

## 6 Conclusion

In this study, we developed two self-scaling conjugate gradient (CG) parameters for solving nonmonotone nonlinear equations. The formulas were derived by integrating the renowned Barzilai-Borwein approach. The proposed algorithms ensure a sufficient descent property, independent of the accuracy of the line search procedure, and guarantee global convergence under appropriate assumptions. Furthermore, we numerically investigate the robustness and computational efficiency of the proposed methods by conducting experiments on benchmark test problems. Their performance is compared against existing methods. The results of these experiments demonstrate that the proposed methods outperform the alternatives based on the adopted comparison metrics. To validate the approach, the discrete kinematic equations of a 3DOF robotic arm were embedded within an optimization framework. This embedding minimized objective functions representing joint trajectory errors. Numerical simulations demonstrated that specific self-scaling CG algorithms exhibit superior convergence properties, making them particularly well-suited for solving inverse kinematics problems in robotic arms. By providing empirical validation and detailed analysis, we believe this study makes a significant contributions to advancing optimization techniques in robotics, paving the way for more reliable and efficient robotic systems.
